# Sex Differences in the Relation between Waist Circumference within the Normal Range and Development of Reflux Esophagitis

**DOI:** 10.3390/jcm8010067

**Published:** 2019-01-09

**Authors:** Hyo-Joon Yang, Yoosoo Chang, Soo-Kyung Park, Yoon Suk Jung, Jung Ho Park, Dong Il Park, Seungho Ryu, Chong Il Sohn

**Affiliations:** 1 Division of Gastroenterology, Department of Internal Medicine and Gastrointestinal Cancer Center, Kangbuk Samsung Hospital, Sungkyunkwan University School of Medicine, Seoul 03181, Korea; hyojoon.yang@samsung.com or hyojoonyang@gmail.com (H.-J.Y.); sk0103.park@samsung.com (S.-K.P.); ys810.jung@samsung.com (Y.S.J.); jungho3.park@samsung.com (J.H.P.); diksmc.park@samsung.com (D.I.P.); 2 Center for Cohort Studies, Total Healthcare Center, Kangbuk Samsung Hospital, Sungkyunkwan University School of Medicine, Seoul 04514, Korea; yoosoochang@gmail.com or yoosoo.chang@gmail.com; 3 Department of Occupational and Environmental Medicine, Kangbuk Samsung Hospital, Sungkyunkwan University School of Medicine, Seoul 03181, Korea; 4 Department of Clinical Research Design and Evaluation, SAIHST, Sungkyunkwan University, Seoul 06351, Korea

**Keywords:** abdominal obesity, waist circumference, cohort studies, gastroesophageal reflux, esophagitis, peptic

## Abstract

We examined the association of abdominal obesity and waist circumference within normal range with the incidence of reflux esophagitis, separately in men and women. This cohort study involved 142,679 Korean adults without reflux esophagitis, who underwent upper endoscopy at baseline and during follow-up. Waist circumference was categorized into the following quartiles: <80, 80.1–85, 85.1–90, and ≥90.1 cm in men; and <69.3, 69.3–74, 74.1–79.5, and ≥79.6 cm in women. During the 551,877.8 person-years of follow-up, 29,509 participants developed reflux esophagitis. The association between waist circumference quartiles and risk of reflux esophagitis significantly differed with sex (*p* for interaction < 0.001). In men, multivariable-adjusted hazard ratios (HRs) (95% confidence intervals (CIs)) comparing waist circumference quartiles 2, 3, and 4 to the lowest quartile were 1.03 (0.99–1.07), 1.08 (1.04–1.12), and 1.15 (1.10–1.19), respectively. In women, HRs (95% CIs) comparing quartiles 1, 2, and 4 to the 3rd quartile were 1.10 (1.04–1.17), 1.03 (0.98–1.10), and 1.07 (1.01–1.13), respectively. In this large cohort with endoscopic follow-up, the risk of reflux esophagitis increased with increasing waist circumference even within the normal range in men, whereas the risk increased with low normal waist circumference or with abdominal obesity in women, indicating a U-shaped association.

## 1. Introduction

Gastroesophageal reflux disease (GERD) is extremely common, accounting for 9 million outpatient clinic visits in the United States each year [1]. GERD, previously uncommon in Asia, has become prevalent and important in the region as well [2]. Abdominal obesity is an important risk factor for GERD, and is associated with reflux esophagitis, Barrett’s esophagus, and esophageal adenocarcinoma [3,4,5].

Increased waist circumference is associated with anatomical disruption of the gastroesophageal junction, leading to reflux esophagitis [6]. A large waist circumference also promotes acid reflux without apparent loss of lower esophageal sphincter tone [7]. In addition, visceral fat secretes pro-inflammatory cytokines, which might contribute to reflux esophagitis [8]. However, most previous studies have focused on the impact of abdominal obesity on reflux esophagitis; however, it remains unclear whether larger waist circumference within the normal range can predispose individuals to the development of reflux esophagitis.

Epidemiological studies have demonstrated a profound male predominance in the prevalence of reflux esophagitis [9,10]. In an animal study, estrogen appears to explain sex-related differences by attenuating the gastroesophageal reflux disease-related esophageal tissue damage [11]. In particular, several cross-sectional studies in Asians have reported that larger waist circumference was associated with increased presence of reflux esophagitis only in men, not in women [5,12]. Furthermore, two Japanese studies have suggested that the risk of reflux esophagitis may be increased, even in underweight women [13,14]. However, these studies were cross-sectional, which have the limitation of temporal ambiguity between risk factors and outcome. Persons with more severe GERD may be more likely to reduce weight and waist circumference as a result of their disease status. Until now, there are limited studies to address the impact of waist circumference, within the normal range, on the development of reflux esophagitis according to sex.

Therefore, we examined whether abdominal obesity and large waist circumference within the normal range is associated with the risk of incident reflux esophagitis in men and women, separately, in a large cohort of young and middle-aged individuals who participated in a health screening examination program.

## 2. Materials and Methods

### 2.1. Study Population

Materials and the Kangbuk Samsung Health Study consisted of a cohort of Korean men and women who underwent comprehensive annual or biennial examinations at Kangbuk Samsung Hospital Total Healthcare Centers in Seoul and Suwon, South Korea [15]. Most examinees (over 80%) were employees of various companies, local government organizations, or the spouses of these employees. In South Korea, the Industrial Safety and Health Law requires annual or biennial health screening examinations of all employees, offered free of charge. Upper endoscopy is widely performed as a part of routine comprehensive health examination in Korea [16].

The present analysis included all study participants who underwent upper endoscopy and waist circumference measurement during a health checkup between January 2002 and December 2015, and had at least one follow-up visit until 31 December 2016 (*n* = 185,462). In the present study, 42,783 individuals were excluded at baseline for the following reasons: missing data on body mass index (BMI) (*n* = 5326); history of gastric or esophageal surgery (*n* = 412); history of malignancy (*n* = 3213); gastric or duodenal ulcer on endoscopy (*n* = 8611); use of gastrointestinal medication (*n* = 9542); or reflux esophagitis at baseline (*n* = 25,007). Since some individuals met multiple exclusion criteria, a total of 142,679 eligible subjects without reflux esophagitis at baseline were included.

This study was approved by the Institutional Review Board of Kangbuk Samsung Hospital (KBSMC 2017-02-015). The requirement for informed consent was waived because only de-identified data obtained as part of routine health screening examinations were used.

### 2.2. Measurements

Baseline and follow-up examinations were conducted at Kangbuk Samsung Hospital Total Healthcare Screening Center clinics. At each clinic visit, information regarding demographic characteristics, health behaviors, and medication use was collected by standardized, self-administered questionnaires. Smoking status was categorized into never, former, and current, and alcohol consumption into none, moderate (<20 g/day), and high (≥20 g/day). The weekly frequency of moderate or vigorous physical activity was also assessed. Education level was categorized as less than college graduate vs. college graduate or more.

The fasting blood sample measurements included levels of glucose, total cholesterol, low-density lipoprotein-cholesterol (LDL-C), triglycerides, high-density lipoprotein-cholesterol (HDL-C), insulin, and high-sensitivity C-reactive protein (hsCRP). Insulin resistance was assessed using the homeostatic model assessment-insulin resistance (HOMA-IR) equation as follows: fasting blood insulin (uU/mL) × fasting blood glucose (mmol/L)/22.5.

Waist circumference, weight, height, and seated blood pressure were measured by trained nurses. Height was measured to the nearest 0.1 cm using a stadiometer with the examinee standing without shoes. Weight was measured to the nearest 0.1 kg in a light gown while standing barefoot, using a bioimpedance analyzer (Inbody 720, Biospace Co., Seoul, Korea), which was calibrated every day before beginning the tests. Obesity was defined as a BMI ≥ 25 kg/m^2^ according to Asian-specific criteria [17]. Waist circumference was measured to the nearest 0.1 cm at the midpoint between the bottom of the rib cage and the top of the iliac crest with the subjects standing, their weight equally distributed on both feet, their arms at their sides, and head facing straight forward. Abdominal obesity was defined as waist circumference ≥90 cm for men and ≥85 cm for women—values which are specific for Korean populations [18,19]. Hypertension was defined either as systolic blood pressure ≥140 mmHg, diastolic blood pressure ≥90 mmHg, or the use of antihypertensive medication. Diabetes mellitus was defined as a fasting serum glucose level ≥126 mg/dL, or current use of anti-diabetic medication.

Upper endoscopy was performed by 13 experienced endoscopists with the Evis Lucera CV-260 endoscope (Olympus Medical Systems, Tokyo, Japan). Reflux esophagitis was defined as mucosal breaks or minimal changes, such as erythema and/or whitish discoloration, and was graded according to the Los Angeles (LA) classification with Japanese modification, from grades M to D [20].

### 2.3. Statistical Analysis

All analyses were performed separately in men and women, as the association between waist circumference and reflux esophagitis has been reported differently according to sex. Waist circumference was categorized into the sex-specific quartiles based on distribution within the study population as follows: 56.5–80, 80.1–85, 85.1–90, 90.1–140.1 for men; 50.9–69.2, 69.3–74.0, 74.1–79.5, 79.6–141.1 for women. Follow-up for each participant extended from the baseline examination until the development of reflux esophagitis or the last health examination conducted prior to 31 December 2016. Incidence rates were calculated as the number of incident cases divided by person-years of follow-up. Since we knew that the onset of reflux esophagitis had occurred at some point between the two visits, but did not know the precise timing, we used a parametric proportional hazards model to take into account this type of interval censoring [21]. In these models, the baseline hazard function was parameterized with restricted cubic splines in log time with four degrees of freedom. We estimated adjusted hazard ratios (HRs) with 95% confidence intervals (CIs) for incident reflux esophagitis, comparing each category of waist circumference to the quartile with lowest risk as the reference. The proportional hazards assumption was assessed by examining the estimated log (−log(survival)) graphs; no violation of the assumption was found.

Models were initially adjusted for age and then further adjusted for potential confounding factors of center (Seoul or Suwon), year of screening exam, smoking status (never, past, current, or unknown), alcohol intake (0, <20, ≥20 g/day, or unknown), physical activity (<3, ≥3 times/week, or unknown), education level (high school graduate or less, community college or university graduate, graduate school or higher, or unknown), history of diabetes (no, yes, or unknown), medication for diabetes (no, yes, or unknown), history of hypertension (no, yes, or unknown), medication for hypertension (no, yes, or unknown) and medication for dyslipidemia (Model 1). Model 2 was further adjusted for BMI (continuous). The association between waist circumference and risk of reflux esophagitis was evaluated below the level of abdominal obesity. Additionally, we performed the analyses in non-obese individuals with a BMI of <25 kg/m^2^. To determine linear risk trends, the number of quartiles was used as a continuous variable and tested in each model. To further explore the shape of the dose–response relationship of waist circumference levels with the development of reflux esophagitis, restricted cubic splines with knots were used at the 5th, 27.5th, 50th, 72.5th, and 95th percentiles of waist circumference distribution.

All analyses were performed using STATA, version 15.0 (StataCorp LP, College Station, TX, USA).

## 3. Results

The mean (standard deviation) age, BMI, and waist circumference of 77,245 male participants at baseline were 39.0 (8.1) years, 24.4 (2.8) kg/m^2^, and 85.1 (7.6) cm, respectively (Table 1). Among 65,434 female participants, the mean (standard deviation) age, BMI, and waist circumference at baseline were 38.7 (8.3) years, 21.7 (3.0) kg/m^2^, and 74.9 (7.9) cm, respectively (Table 2). In men, waist circumference was positively associated with age, obesity, current smoking, alcohol intake, diabetes, hypertension, medication for dyslipidemia, higher levels of BMI, blood pressures, glucose, total cholesterol, LDL-C, triglycerides, HOMA-IR, and hsCRP; and negatively associated with regular exercise, education level, and HDL-C. In women, waist circumference was positively associated with age, obesity, alcohol intake, regular exercise, diabetes, hypertension, medication for dyslipidemia, higher levels of BMI, blood pressures, glucose, total cholesterol, LDL-C, triglycerides, HOMA-IR, and hsCRP; and negatively associated with education level and HDL-C.

Table 3 shows the relationship between waist circumference quartiles and incident reflux esophagitis. Over 551,877.8 person-years of follow-up, 29,509 participants developed reflux esophagitis (incidence density, 53.5 per 1000 person-years in overall; 64.1 per 1000 person-years in men; and 40.3 per 1000 person-years in women) (Table 3). The median follow-up period was 3.1 years (interquartile range, 2.0–5.1; maximum, 14.7 years). The association between waist circumference quartiles and risk of reflux esophagitis significantly differed according to sex (*p* for interaction < 0.001). Increasing quartiles of waist circumference were positively associated with risk of reflux esophagitis in men, whereas a U-shaped association between waist circumference and reflux esophagitis was observed in women. After adjusting for age, screening center, year of screening examination, smoking status, alcohol intake, regular exercise, educational level, history of diabetes, medication for diabetes, history of hypertension, medication for hypertension, and medication for dyslipidemia, multivariable-adjusted HRs (95% CIs) comparing waist circumference quartiles 2, 3, and 4 to the lowest quartile were 1.03 (0.99–1.07), 1.08 (1.04–1.12), and 1.15 (1.10–1.19), respectively, among men, whereas multivariable-adjusted HRs (95% CIs) comparing waist circumference quartiles 1, 2, and 4 to the 3rd quartile, with the lowest risk as the reference category among women, were 1.10 (1.04–1.17), 1.03 (0.98–1.10), and 1.07 (1.01–1.13), respectively. These associations were virtually unchanged after further adjustment for BMI.

**Figure 1:**
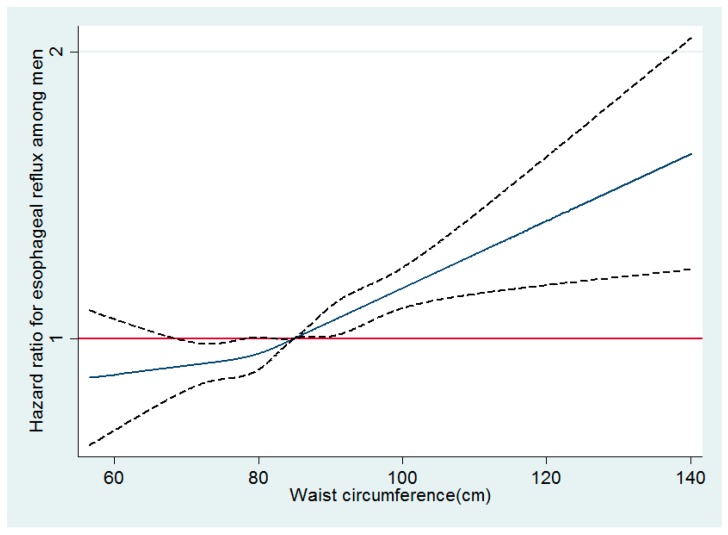
Multivariable-adjusted hazard ratios for reflux esophagitis by waist circumference among men. Curves represent adjusted hazard ratios for reflux esophagitis based on restricted cubic splines with knots at the 5th, 27.5th, 50th, 72.5th, and 95th percentiles of waist circumference. Models were adjusted for age, center, year of screening exam, smoking status, alcohol intake, physical activity, education level, history of diabetes, medication for diabetes, history of hypertension, medication for hypertension, and medication for dyslipidemia. The red solid line represents the reference plane; the navy solid line represents hazard ratio; and the black dotted lines represent 95% confidence intervals.

**Figure 2:**
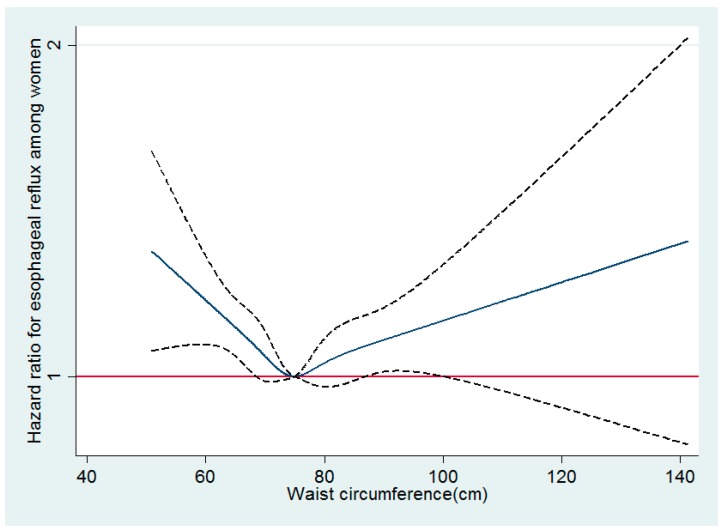
Multivariable-adjusted hazard ratios for reflux esophagitis by waist circumference among women. Curves represent adjusted hazard ratios for reflux esophagitis based on restricted cubic splines with knots at the 5th, 27.5th, 50th, 72.5th, and 95th percentiles of waist circumference. Models were adjusted for age, center, year of screening exam, smoking status, alcohol intake, physical activity, education level, history of diabetes, medication for diabetes, history of hypertension, medication for hypertension, and medication for dyslipidemia. The red solid line represents the reference plane; the navy solid line represents hazard ratio; and the black dotted lines represent 95% confidence intervals.

In multivariable-adjusted spline regression models of both men and women, a linear association between waist circumference and reflux esophagitis was observed in men, while a U-shaped association between waist circumference and reflux esophagitis was observed around 75 cm as a reflection point in women (Figure 1 and Figure 2).

Table 4 shows the risk of reflux esophagitis according to abdominal obesity and waist circumference quartiles within normal range. In model 1, multivariable-adjusted HRs (95% CIs) comparing waist circumference quartiles 2, 3, 4, and abdominal obesity to the lowest quartile were 1.05 (1.00–1.10), 1.05 (1.01–1.10), 1.09 (1.04–1.15) and 1.16 (1.11–1.21), respectively, among men, whereas, among women, HRs (95% CIs) comparing waist circumference quartiles 1, 2, 4, and abdominal obesity to the 3rd quartile were 1.10 (1.04–1.17), 1.02 (0.96–1.08), 1.04 (0.98–1.11), and 1.07 (0.99–1.15), respectively. Further adjustment for BMI slightly attenuated these associations but remained significant.

We also looked for the association between waist circumference and risk of reflux esophagitis among non-obese individuals with BMI <25 kg/m^2^. After adjusting for age, screening center, year of screening examination, smoking status, alcohol intake, regular exercise, educational level, history of diabetes, medication for diabetes, history of hypertension, medication for hypertension, and medication for dyslipidemia, multivariable-adjusted HRs (95% CIs) comparing waist circumference quartiles 2, 3, and 4 to the lowest quartile were 1.02 (0.98–1.07), 1.08 (1.03–1.14), and 1.18 (1.06–1.30), respectively, among non-obese men with BMI of <25 kg/m^2^ (*p* for linear trend < 0.001). For women, multivariable-adjusted HRs (95% CIs) comparing waist circumference quartiles 1, 2, and 4 to the 3rd quartile were 1.10 (1.03–1.16), 1.03 (0.97–1.09), and 1.06 (0.99–1.14), respectively, among women with BMI of <25 kg/m^2^ (*p* for quadratic term = 0.001).

## 4. Discussion

The present study demonstrated sex-specific association between a wide range of waist circumferences and incident reflux esophagitis in a large cohort of Korean population. We found a positive and graded association between waist circumference and the risk of incident reflux esophagitis among men even in the normal range of waist circumference, suggesting that the risk of reflux esophagitis rises with waist circumference with both normal and abnormal range waist circumferences. In addition, the association between increasing quartiles of waist circumference and risk of reflux esophagitis was similarly observed in non-obese men with BMI of <25 kg/m^2^. In women, the risk of reflux esophagitis was increased in low normal waist circumferences as well as abdominal obesity, indicating a U-shape association between waist circumference and reflux esophagitis. The associations remained significant after controlling for multiple potential confounding variables, including BMI and metabolic parameters.

A recent systematic review demonstrated that abdominal obesity is associated with reflux esophagitis [4]. However, most of the included studies and, importantly, all three cohort studies have focused on abdominal obesity while using participants with normal waist circumference as the reference group; therefore, they could not find the impact of levels of waist circumference within the normal range on the risk of reflux esophagitis [22,23,24]. Our study adds new information that the risk of reflux esophagitis appears to increase across the full range of waist circumference in men while considering both normal and abnormal levels of waist circumference. Moreover, this association was observed even in non-obese men. This suggests that a moderate increase in waist circumference, even among non-obese men with a normal level of waist circumference, may increase the risk for the development of reflux esophagitis. This is of particular concern given the persistently high prevalence of abdominal obesity in children and adolescents in the United States [25] and, recently, increasing prevalence in young Asian adults [26,27].

The association between abdominal obesity and the risk of reflux esophagitis among Asian women remains controversial. In a cross-sectional study from the Kaiser Permanente multiphasic health checkup cohort in the United States, abdominal obesity measured by sagittal abdominal diameter was not associated with reflux symptoms in Asian men and women, although sample size was relatively small for Asians [3]. Two cross-sectional studies from Korea and Japan have shown that the waist circumference is associated with reflux esophagitis only in men, but not in women [5,12]; however, two studies were limited by a small number of women with reflux esophagitis in the study cohort (only 63 and 20, respectively). In the present study, we included a large number of 9763 incident cases among 68,434 female participants, and demonstrated that women with low normal waist circumference, as well as women with abdominal obesity, had an increased risk of reflux esophagitis compared to women with mid normal waist circumference showing a U-shaped association between waist circumference and the risk of reflux esophagitis.

A causative mechanism for increased risk of reflux esophagitis in women with low normal waist circumference, seen in our study, is incompletely understood. Abdominal compression by tight clothing can be a possible explanation. It was demonstrated that acid reflux can be promoted with elevated intra-abdominal pressure by tight waist belt [28,29,30]. Young women who are exposed to social media are more conscious about their appearance and likely to perceive a slim body as attractive [31], for which they may attempt to lose weight and wear tight garments, which may lead to development or aggravation of reflux. Decreased estrogen effect by low body fat mass may be another explanation. In a rat model, estrogen reduced esophageal tissue damage by exogenous nitric oxide exposure [11]. Furthermore, underweight or very low waist circumference may reflect sarcopenia, which has been reported to increase the risk of reflux esophagitis [32]. Further studies with assessment of body composition and clothing pattern are required to elucidate the mechanism underlying the association between low waist circumference and an increased risk of reflux esophagitis.

The strengths of our study include the large sample size of each sex, cohort study design, and endoscopic evaluation at baseline and follow-up visits, which enabled accurate evaluation of the effect of waist circumference, within normal range, on the future risk of reflux esophagitis. The relatively young age of our cohort participants compared to others would be another advantage because our results have implications in young men with high normal waist circumference for future risk of reflux esophagitis on relatively short-term follow-up, and long-standing reflux esophagitis since young adulthood, which may be another concern as it implies increased risk of more serious complications of GERD, such as Barrett’s esophagus and esophageal adenocarcinoma in the future.

This study also has some limitations. First, due to a relatively long study duration (up to 14.7 years), different examiners, nurses, and technicians were involved in performing health screening exams over time. However, study personnel collecting the data were unaware of the study aims, and changes in examiners and equipment were independent of participant characteristics. Second, information on dietary factors, which could be associated with both waist circumference and reflux esophagitis, was not available in this study. Finally, our findings from relatively healthy young and middle-aged Korean men and women who participated in company- or organization-paid health checkups may not be representative of other populations of different age and race/ethnicity or populations in different settings.

## 5. Conclusions

In this large cohort with endoscopic follow-up, the risk of reflux esophagitis increased with increasing waist circumferences even within normal range in men, whereas the risk increased in women with low normal waist circumferences or abdominal obesity, indicating a U-shaped association. Further studies are required to elucidate the mechanism underlying sex differences in the association between waist circumference and the risk of reflux esophagitis.

## Figures and Tables

**Table 1:** Baseline characteristics of study participants by waist circumference among men.

Characteristics	Overall	Quartiles of Waist Circumference (cm)				*p* Value
		Quartile 1 (56.5–80)	Quartile 2 (80.1–85)	Quartile 3 (85.1–90)	Quartile 4 (90.1–140.1)	
Number	77,245	20,065	20,239	18,855	18,086	<0.001
Age (years) ^1^	39.0 (8.1)	37.8 (7.8)	39.0 (8.1)	39.7 (8.3)	39.5 (8.2)	<0.001
Obesity (%)	38.8	1.8	17.2	51.4	90.9	<0.001
Current smoker (%)	36.5	34.9	34.8	37.1	39.8	<0.001
Alcohol intake (%) ^2^	30.0	22.6	28.4	32.4	37.2	<0.001
Vigorous exercise (%) ^3^	15.8	16.9	16.6	15.4	14.0	<0.001
High education level (%) ^4^	88.8	89.4	89.4	88.5	87.7	<0.001
Diabetes (%)	3.9	1.9	3.2	4.3	6.4	<0.001
Hypertension (%)	16.0	8.5	13.1	17.7	25.7	<0.001
Medication for dyslipidemia (%)	2.0	0.8	1.6	2.1	3.4	<0.001
BMI (kg/m^2^)	24.4 (2.8)	21.6 (1.7)	23.6 (1.4)	25.1 (1.5)	27.7 (2.3)	<0.001
Systolic BP (mmHg) ^1^	115.1 (11.7)	111.7 (11.1)	114.1 (11.3)	115.8 (11.4)	119.1 (11.7)	<0.001
Diastolic BP (mmHg) ^1^	74.7 (9.1)	72.4 (8.5)	74.0 (8.8)	75.3 (9.0)	77.3 (9.2)	<0.001
Glucose (mg/dL) ^1^	95.5 (15.3)	92.3 (13.1)	94.8 (14.1)	96.5 (15.2)	98.9 (18.0)	<0.001
Uric acid (mg/dL) ^1^	6.2 (1.2)	5.8 (1.1)	6.1 (1.2)	6.3 (1.2)	6.5 (1.3)	<0.001
Total cholesterol (mg/dL) ^1^	198.1 (33.8)	188.2 (31.4)	197.6 (32.9)	202.1 (34.2)	205.3 (34.5)	<0.001
LDL-C (mg/dL) ^1^	123.1 (30.5)	122.6 (28.3)	122.8 (29.6)	126.9 (30.3)	130.8 (31.1)	<0.001
HDL-C (mg/dL) ^1^	52.6 (12.0)	57.6 (12.8)	53.1 (11.7)	50.7 (10.9)	48.3 (10.3)	<0.001
Triglycerides (mg/dL) ^5^	114 (81–164)	87 (66–119)	110 (80–154)	126 (90–178)	144 (104–201)	<0.001
ALT (U/L) ^5^	24 (18–35)	19 (15–25)	23 (17–31)	27 (20–37)	33 (24–48)	<0.001
HOMA-IR ^5^	1.58 (1.03–2.23)	1.24 (0.75–1.76)	1.47 (0.96–2.04)	1.68 (1.15–2.33)	2.05 (1.43–2.89)	<0.001
hsCRP (mg/L) ^5^	0.5 (0.3–1.0)	0.3 (0.2–0.6)	0.5 (0.3–0.9)	0.6 (0.3–1.1)	0.8 (0.5–1.5)	<0.001

Data are ^1^ means (standard deviation), ^5^ medians (interquartile range), or percentages. ^2^ ≥20 g of ethanol per day; ^3^ ≥3 time per week; ^4^ ≥college graduate. ALT, alanine aminotransferase; BMI, body mass index; BP, blood pressure; HDL-C, high-density lipoprotein-cholesterol; hsCRP, high sensitivity C-reactive protein; HOMA-IR, homeostasis model assessment of insulin resistance; LDL-C, low-density lipoprotein-cholesterol.

**Table 2:** Baseline characteristics of study participants by waist circumference among women.

Characteristics	Overall	Quartiles of Waist Circumference (cm)				*p* Value
		Quartile 1 (50.9–69.2)	Quartile 2 (69.3–74.0)	Quartile 3 (74.1–79.5)	Quartile 4 (79.6–141.2)	
Number	65,434	16,423	16,778	16,061	16,172	<0.001
Age (years) ^1^	38.7 (8.3)	36.1 (6.6)	37.7 (7.2)	39.3 (8.1)	41.8 (9.9)	<0.001
Obesity (%)	12.5	<0.001	0.6	5.0	44.8	<0.001
Current smoker (%)	3.0	3.1	2.9	2.7	3.2	0.930
Alcohol intake (%) ^2^	4.4	3.2	4.4	4.4	5.8	<0.001
Vigorous exercise (%) ^3^	13.5	12.0	13.2	13.9	14.9	<0.001
High education level (%) ^4^	75.8	83.2	79.6	75.0	65.4	<0.001
Diabetes (%)	1.6	0.4	0.7	1.3	3.9	<0.001
Hypertension (%)	5.9	1.9	3.0	5.8	13.1	<0.001
Medication for dyslipidemia (%)	1.2	0.3	0.7	1.0	2.9	<0.001
BMI (kg/m^2^)	21.7 (3.0)	19.2 (1.5)	20.7 (1.5)	22.0 (1.7)	25.1 (2.9)	<0.001
Systolic BP (mmHg) ^1^	104.1 (12.4)	101.2 (10.9)	102.4 (11.5)	104.2 (12.2)	108.8 (13.5)	<0.001
Diastolic BP (mmHg) ^1^	66.9 (8.9)	65.4 (8.0)	66.0 (8.5)	66.9 (8.9)	69.4 (9.6)	<0.001
Glucose (mg/dL) ^1^	90.8 (11.4)	88.1 (8.4)	89.5 (8.9)	91.0 (10.6)	94.7 (15.4)	<0.001
Uric acid (mg/dL) ^1^	4.2 (0.9)	4.0 (0.8)	4.1 (0.8)	4.2 (0.8)	4.5 (0.9)	<0.001
Total cholesterol (mg/dL) ^1^	186.2 (32.1)	179.8 (28.8)	182.9 (30.5)	187.4 (32.1)	195.1 (34.6)	<0.001
LDL-C (mg/dL) ^1^	107.4 (28.6)	97.9 (24.1)	103.3 (26.5)	109.6 (28.2)	119.1 (30.7)	<0.001
HDL-C (mg/dL) ^1^	63.4 (14.2)	68.2 (14.1)	65.3 (13.9)	62.3 (13.7)	57.8 (13.1)	<0.001
Triglycerides (mg/dL) ^5^	72 (55–97)	62 (50–79)	67 (53–89)	73 (57–98)	90 (66–126)	<0.001
ALT (U/L) ^5^	14 (11–18)	13 (10–17)	13 (10–17)	14 (11–18)	16 (12–23)	<0.001
HOMA-IR ^5^	1.34 (0.83–1.93)	1.20 (0.72–1.72)	1.21 (0.76–1.76)	1.31 (0.84–1.90)	1.66 (1.10–2.39)	<0.001
hsCRP (mg/L) ^5^	0.3 (0.2–0.6)	0.2 (0.1–0.4)	0.2 (0.2–0.5)	0.3 (0.2–0.6)	0.6 (0.3–1.2)	<0.001

Data are ^1^ means (standard deviation), ^5^ medians (interquartile range), or percentages. ^2^ ≥20 g of ethanol per day; ^3^ ≥3 time per week; ^4^ ≥college graduate. ALT, alanine aminotransferase; BMI, body mass index; BP, blood pressure; HDL-C, high-density lipoprotein-cholesterol; hsCRP, high sensitivity C-reactive protein; HOMA-IR, homeostasis model assessment of insulin resistance; LDL-C, low-density lipoprotein-cholesterol.

**Table 3:** Development of reflux esophagitis according to waist circumference quartiles by sex.

Quartiles of Waist Circumference (cm)	Person-Years	Incident Case	Incidence Density (Per 1000 Person-Years)	Age-Adjusted HR (95% CI)	Multivariate HR ^1^ (95% CI)	
					Model 1	Model 2
Men						
Quartile 1 (56.5-80)	86,074.6	5027	58.4	1.00 (reference)	1.00 (reference)	1.00 (reference)
Quartile 2 (80.1-85)	81,053.4	4967	61.3	1.08 (1.04–1.13)	1.03 (0.99–1.07)	1.02 (0.98–1.07)
Quartile 3 (85.1-90)	72,998.0	4777	65.4	1.17 (1.13–1.22)	1.08 (1.04–1.12)	1.07 (1.02–1.12)
Quartile 4 (90.1-140.1)	64,878.3	4790	73.8	1.34 (1.29–1.40)	1.15 (1.10–1.19)	1.13 (1.06–1.21)
*p* for trend				<0.001	<0.001	<0.001
Women						
Quartile 1 (50.9–69.2)	68,561.4	2723	39.7	0.91 (0.86–0.96)	1.10 (1.04–1.17)	1.07 (1.01–1.14)
Quartile 2 (69.3–74.0)	64,214.7	2530	39.4	0.95 (0.90–1.01)	1.03 (0.98–1.10)	1.02 (0.96–1.08)
Quartile 3 (74.1–79.5)	58,297.0	2309	39.6	1.00 (reference)	1.00 (reference)	1.00 (reference)
Quartile 4 (79.6–141.2)	55,800.4	2386	42.8	1.15 (1.09–1.22)	1.07 (1.01–1.13)	1.10 (1.03–1.17)
*p* for quadratic				0.046	<0.001	0.002

*p* < 0.001 for the overall interaction between sex and quartiles of waist circumference for reflux esophagitis (adjusted model 1). ^1^ Estimated from parametric proportional hazards models. Multivariable model 1 was adjusted for age, center, year of screening exam, smoking status, alcohol intake, physical activity, education level, history of diabetes, medication for diabetes, history of hypertension, medication for hypertension and medication for dyslipidemia: Model 2: model 1 plus adjustment for BMI. BMI, body mass index; CI, confidence intervals; HR, hazard ratios.

**Table 4:** Development of reflux esophagitis according to abdominal obesity and waist circumference quartiles within normal range by sex.

Quartiles of Waist Circumference (cm)	Person-Years	Incident Case	Incidence Density (Per 1000 Person-Years)	Age-Adjusted HR (95% CI)	Multivariate HR ^1^ (95% CI)	
					Model 1	Model 2
Men						
Q1 (56.5~78.3)	62,817.9	3610	57.5	1.00 (reference)	1.00 (reference)	1.00 (reference)
Q2 (78.4~82.5)	59,991.6	3686	61.4	1.10 (1.05–1.15)	1.05 (1.00–1.10)	1.04 (0.99–1.10)
Q3 (82.6~86.0)	60,832.1	3780	62.1	1.12 (1.07–1.18)	1.05 (1.01–1.10)	1.05 (0.99–1.10)
Q4 (86.1~89.9)	51,213.0	3375	65.9	1.21 (1.15–1.27)	1.09 (1.04–1.15)	1.08 (1.02–1.15)
Abdominal obesity (≥90)	70,149.7	5110	72.8	1.35 (1.30–1.41)	1.16 (1.11–1.21)	1.14 (1.06–1.22)
*p* for trend				<0.001	<0.001	<0.001
Women						
Q1 (50.9~68.8)	61,497.0	2467	40.1	0.93 (0.87–0.98)	1.10 (1.04–1.17)	1.06 (1.00–1.14)
Q2 (68.9~73.0)	58,559.5	2266	38.7	0.94 (0.89–0.99)	1.02 (0.96–1.08)	1.01 (0.95–1.07)
Q3 (73.1~77.4)	51,177.0	2024	39.5	1.00 (reference)	1.00 (reference)	1.00 (reference)
Q4 (77.5~84.9)	51,625.9	2137	41.4	1.10 (0.98–1.16)	1.04 (0.98–1.11)	1.06 (1.00–1.13)
Abdominal obesity (≥85)	24,014.2	1054	43.9	1.22 (0.99–1.33)	1.07 (0.99–1.15)	1.14 (1.03–1.25)
*p* for quadratic				<0.001	0.001	0.004

*p* < 0.001 for the overall interaction between sex and quartiles of waist circumference for reflux esophagitis (adjusted model 1). ^1^ Estimated from parametric proportional hazards models. Multivariable model 1 was adjusted for age, center, year of screening exam, smoking status, alcohol intake, physical activity, education level, history of diabetes, medication for diabetes, history of hypertension, medication for hypertension, and medication for dyslipidemia: Model 2: model 1 plus adjustment for BMI. BMI, body mass index; CI, confidence intervals; HR, hazard ratios.
